# Gram-negative bacteremia induces greater magnitude of inflammatory response than Gram-positive bacteremia

**DOI:** 10.1186/cc8898

**Published:** 2010-03-04

**Authors:** Ryuzo Abe, Shigeto Oda, Tomohito Sadahiro, Masataka Nakamura, Yo Hirayama, Yoshihisa Tateishi, Koichiro Shinozaki, Hiroyuki Hirasawa

**Affiliations:** 1 Department of Emergency and Critical Care Medicine, Chiba University Graduate School of Medicine, 1-8-1 Inohana Chuo, Chiba, 260-8677, Japan

## Abstract

**Introduction:**

Bacteremia is recognized as a critical condition that influences the outcome of sepsis. Although large-scale surveillance studies of bacterial species causing bacteremia have been published, the pathophysiological differences in bacteremias with different causative bacterial species remain unclear. The objective of the present study is to investigate the differences in pathophysiology and the clinical course of bacteremia caused by different bacterial species.

**Methods:**

We reviewed the medical records of all consecutive patients admitted to the general intensive care unit (ICU) of a university teaching hospital during the eight-year period since introduction of a rapid assay for interleukin (IL)-6 blood level to routine ICU practice in May 2000. White blood cell count, C-reactive protein (CRP), IL-6 blood level, and clinical course were compared among different pathogenic bacterial species.

**Results:**

The 259 eligible patients, as well as 515 eligible culture-positive blood samples collected from them, were included in this study. CRP, IL-6 blood level, and mortality were significantly higher in the septic shock group (n = 57) than in the sepsis group (n = 127) (*P *< 0.001). The 515 eligible culture-positive blood samples harbored a total of 593 isolates of microorganisms (Gram-positive, 407; Gram-negative, 176; fungi, 10). The incidence of Gram-negative bacteremia was significantly higher in the septic shock group than in the sepsis group (*P *< 0.001) and in the severe sepsis group (n = 75, *P *< 0.01). CRP and IL-6 blood level were significantly higher in Gram-negative bacteremia (n = 176) than in Gram-positive bacteremia (n = 407) (*P *< 0.001, <0.0005, respectively).

**Conclusions:**

The incidence of Gram-negative bacteremia was significantly higher in bacteremic ICU patients with septic shock than in those with sepsis or severe sepsis. Furthermore, CRP and IL-6 levels were significantly higher in Gram-negative bacteremia than in Gram-positive bacteremia. These findings suggest that differences in host responses and virulence mechanisms of different pathogenic microorganisms should be considered in treatment of bacteremic patients, and that new countermeasures beyond conventional antimicrobial medications are urgently needed.

## Introduction

Despite recent advances in critical care medicine, the mortality of sepsis in ICU remains high [1,2]. Among various infections underlying sepsis, bacteremia is recognized as a critical condition that influences the outcome of sepsis [3,4], and is reportedly associated with an attributable mortality of approximately 35% [5]. While the larger part of pathogens in sepsis-inducing infections was previously Gram-negative bacteria, currently the larger part of pathogens identified in sepsis is Gram-positive bacteria [1,6], with an increasing proportion of multi-resistant bacteria [7]. Although large-scale surveillance studies of bacterial species causing bacteremia have been published [7,8], the pathophysiological differences in bacteremias with different causative bacterial species remain unclear.

Since 2000, we have used a rapid assay system for interleukin (IL)-6 blood level, aiming at real-time assessment of the magnitude of inflammatory response to facilitate prompt determination of disease severity and therapeutic effects [9]. The objective of the present study was to investigate differences in the pathophysiology and clinical course of bacteremia caused by different bacterial species by cross-check review of laboratory findings and the clinical record with pathogenic microbial species in bacteremic patients who were admitted to the ICU during the eight years since introduction of the rapid IL-6 assay to routine ICU practice.

## Materials and methods

### Study population

**Figure 1:**
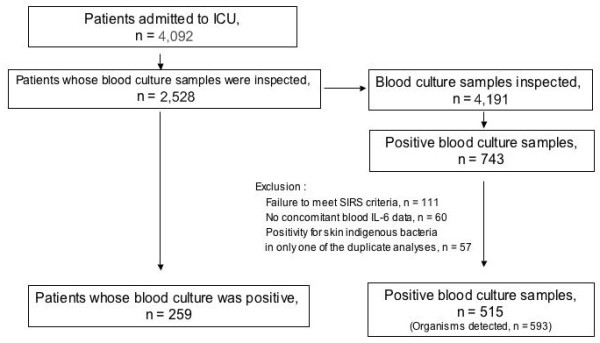
**Selection of eligible patients and blood culture samples**. Patients were admitted to the ICU between May 2000 and October 2008. SIRS: systemic inflammatory response syndrome.

We reviewed the medical records of all consecutive patients admitted to the general ICU of a university teaching hospital during the period from May 2000 to October 2008. Patients with one or more blood samples processed for culture were enrolled in the study. Among culture-positive patients, those fulfilling diagnostic criteria for sepsis described below and undergoing blood sampling for measurement of white blood cell count (WBC), C-reactive protein (CRP), and IL-6 concomitantly with collection of blood culture samples were finally included in the extensive review described below (see Figure 1). Informed consent for blood sampling as a part of daily practice and later use of the data for academic purpose was obtained from all patients or their family members when the patients were admitted to the ICU. The study was approved by the institutional ethics committee.

For the diagnosis of sepsis, the criteria of the American College of Chest Physicians/Society of Critical Care Medicine Consensus Conference were applied [10]. The criteria were as follows. Fulfillment of both of the following, (1) and (2), was required: (1) The presence of systemic inflammatory response syndrome (manifested by two or more of the following criteria: fever (temperature above 38°C) or hypothermia (temperature below 35.5°C), tachycardia (more than 90 beats per minute), tachypnea (more than 20 breaths per minute), or hypocapnia (PaCO2 of less than 32 torr), and leukocytosis or leukopenia (white blood cell count of more than 12,000/mm^3 ^or less than 4,000/mm^3^, respectively)); (2) a documented source of infection.

Among patients meeting the diagnostic criteria for sepsis described above, those also meeting at least one of the following criteria for organ failure were classified in the severe sepsis group: hypoxemia (PaO2/FiO2 < 300), acute oliguria (urine output <0.5 mL/kg/hr persisting two hours or longer), serum creatinine >2.0 mg/dL, coagulation disorder (PT-INR > 1.5), thrombocytopenia (PLT < 100,000/mL), hyperbilirubinemia (T-Bil > 2.0 mg/dL), and hyperlactatemia (blood lactate >18 mg/dL). Of those in the severe sepsis group, those with a systolic pressure of 90 mmHg or lower that persisted despite appropriate fluid resuscitation and required a vasopressor were classified in the septic shock group. The remaining patients classified in neither the *severe sepsis *nor the *septic shock *group comprised the sepsis group.

Patients with hematological malignancies and autoimmune disorders who needed treatment of immunosuppressive drug therapy were excluded from the present study. Immunosuppressive drugs include predonisolone, methylpredonisolone, cyclophosphamide, cyclosporine, doxorubicin, vincristine, methotrexate, rituximab and FK506. Patients with positive blood culture but not meeting the diagnostic criteria for sepsis were also excluded from the study to eliminate the possibility of samples false-positive as a result of contamination. Samples collected through central venous catheter and samples collected through peripheral vein puncture were excluded from the study to eliminate the possible variance of blood levels of biomarkers between arterial blood and venous blood. Patients whose blood culture showed skin indigenous bacteria in only one of the duplicate samples were also excluded. Coagulase-negative Staphylococcus, Corynebacterium species, Micrococcus species and Propionibacterium species were defined as skin indigenous bacteria.

### Blood culture

Blood culture samples were collected from arterial catheters by ICU staff doctors. Before taking blood samples, catheter ports or stopcocks were disinfected with povidone-iodone swab and 70% isopropyl alcohol swab. A 10 mL blood sample was divided evenly into anaerobic and aerobic culture bottles at the bedside. Blood samples were processed using a BACTEC 9240 automated blood culture system in combination with both standard aerobic and anaerobic media available from the instrument manufacturer (Becton Dickinson Diagnostic Instrument Systems, Paramus, NJ, USA). Bacteria were identified using standard methods. Two distinct episodes of bloodstream infection were recorded for a patient, regardless of bacterial species detected, if at least six days had elapsed between the two positive blood cultures, provided appropriate therapy had been implemented and significant clinical improvement had been obtained between the two episodes.

### Cytokine blood levels

Blood samples were obtained from arterial catheter simultaneously with collection of culture samples in all the patients studied. IL-6 blood levels were measured with a chemiluminescence enzyme immunoassay using a rapid measurement system (Human IL-6 CLEIA, Fujirebio, Tokyo, Japan). The duration of processing for IL-6 measurement was approximately 30 minutes [9].

### Grouping of patients and blood culture samples

First, the three patient groups divided according to severity of sepsis (sepsis, severe sepsis, and septic shock) were compared for white blood count, CRP, and IL-6 blood level as well as mortality.

Culture-positive blood samples were divided into two groups, Gram-positive (GP) sample group and Gram-negative (GN) sample group, according to the bacterial species detected. When both Gram-positive and Gram-negative bacteria were detected in one blood culture sample, the sample was included in both the GP and GN sample groups. WBC, CRP, and IL-6 blood levels were compared between these two sample groups.

Finally, all bacteremic patients were divided into three groups according to bacterial species detected during the clinical course: GP patients' group, one or more Gram-positive species detected; GN patients' group, one or more Gram-negative species detected; and GP/GN patients' group, both Gram-positive and Gram-negative species detected. These three patient groups were compared for severity and clinical outcome. Severity of illness was assessed by calculating Acute Physiology and Chronic Health Evaluation (APACHE)-II score [11] and Sequential Organ Failure Assessment (SOFA) score [12].

### Statistical analysis

Comparisons of variables among groups were performed with the unpaired Student's t-test, except for sex, mortality, and positivity for Gram-positive and Gram-negative bacteria, which were compared with the chi-square test. Statistical significance was defined as *P *< 0.05. Statistical analyses were performed with the SPSS 13.0 J for Windows software package (SPSS Inc, Chicago, IL, USA).

## Results

Between May 2000 and October 2008, 4,092 patients were admitted to the ICU, and 2,528 of them underwent blood culture tests. Positive blood culture was confirmed for 743 of 4,191 samples submitted. After eliminating those meeting the exclusion criteria the remaining 515 culture-positive samples were included in the present study. These samples were collected from 259 patients and harbored a total of 593 microorganism isolates (Figure 1).

The 259 eligible patients included 127 patients in the sepsis group, 75 patients in the severe sepsis group, and 57 patients in the septic shock group. Table 1 summarizes background characteristics, WBC, CRP, and IL-6 (measured concomitantly with collection of culture-positive blood samples) as well as causative microorganisms and mortality in the three patient groups. Results demonstrated that CRP level was significantly higher in the septic shock group than in the sepsis group. The IL-6 blood level was significantly higher in the septic shock group than in the sepsis and in the severe sepsis groups. Furthermore, mortality in the septic shock group was significantly higher than that in the sepsis group. The incidence of Gram-positive bacteremia in the septic shock group was significantly lower than those in the two other patient groups, while the incidence of Gram-negative bacteremia was significantly higher in the septic shock group than in any other group. The incidence of bacteremia caused by both Gram-positive and Gram-negative bacteria was significantly higher in the septic shock group than in the sepsis group; this was also the case for the incidence of bacteremia caused by multiple organisms.

**Table 1:** Patients' characteristics, white blood cell count, C-reactive protein and interleukin-6 blood level and mortality

	Total N = 259	Sepsis N = 127	Severe sepsis N = 75	Septic shock N = 57	*P *value
Age yrs, mean (SD)	58.1 (18.6)	54.7 (18.6)	61.0 (17.3)	61.7 (19.2)	<0.05^a^,^b^
Male, n (%)	180 (69.5)	88 (69.3)	55 (73.3)	37 (64.9)	ns
WBC (*10^3^/mm^3^), mean (SD)	14.0 (9.5)	14.1 (8.1)	15.2 (10.9)	12.8 (11.0)	ns
CRP (mg/dL), mean (SD)	11.8 (9.1)	10.0 (8.5)	11.4 (9.3)	15.6 (9.5)	<0.001^b^
IL-6 (pg/mL), mean (SD)	33,543 (136,974)	8,398 (47,705)	8,176 (37,975)	118,435 (264,819)	<0.001^b^,^c^
Gram positive bacteremia, n (%)	168 (64.9)	92 (72.4)	51 (68.0)	25 (43.9)	<0.0005^d^ <0.01^e^
Gram negative bacteremia, n (%)	70 (27.0)	28 (22.0)	17 (22.7)	25 (43.9)	<0.005^d^ <0.01^e^
Both of Gram positive and negative bacteremia*, n (%)	15 (5.8)	4 (3.1)	4 (5.3)	7 (12.3)	<0.05^d^
Fungemia*, n (%)	7 (2.7)	3 (2.3)	3 (4.0)	1 (1.8)	ns
Bacteremia caused by multiple organisms, n (%)	16 (6.2)	4 (3.1)	4 (5.3)	8 (14.0)	<0.01^d^
Length of ICU stay (day), mean (SD)	19.4 (21.7)	20.6 (22.4)	17.4 (17.7)	19.5 (24.9)	ns
Mortality (%)	31.3	20.5	36.0	49.1	<0.001^d^

WBC, white blood cell count; CRP, C-reactive protein; IL-6, interleukin-6. *One case overlapping because Gram-positive bacteria and fungi were detected. ^a^With unpaired Student's T test, between sepsis group and severe sepsis group. ^b^With unpaired Student's T test, between sepsis group and septic shock group. ^c ^With unpaired Student's T test, between severe sepsis group and septic shock group. ^d ^With Chi square test, between sepsis group and septic shock group. ^e ^With Chi square test, between severe sepsis group and septic shock group.

Table 2 compares patient characteristics, severity scores, length of ICU stay and mortality among GP patients' group (n = 168), GN patients' group (n = 70) and GP/GN patients' group (n = 15). APACHE II score was significantly higher in the GN patients' group than in GP patients' group, while no significant differences were noted between any pair of groups examined.

**Table 2:** Patients' characteristics, severity scores, length of ICU stay and mortality in GP, GN, GP/GN groups

	GP patients' group (*n* = 168)	GN patients' group (*n* = 70)	GP/GN patients' group (*n* = 15)	*P *value
Age (yrs), mean (SD)	56.2 (18.9)	61.7 (17.2)	60.3 (21.0)	ns
Male, *n *(%)	118 (70.2)	48 (68.6)	9 (60.0)	ns
APACHE II, mean (SD)	21.8 (9.5)	24.6 (7.4)	23.6 (10.7)	<0.05^a^
SOFA, mean (SD)	9.53 (5.0)	10.71 (4.4)	11.66 (6.0)	ns
Length of ICU stay (days), mean (SD)	18.7 (15.8)	20.5 (29.6)	16.2 (17.5)	ns
Mortality, (%)	28.0	40.0	33.3	ns

GP, Gram-positive; GN, Gram-negative; GP/GN, Gram-positive and Gram-negative; APACHE-II, Acute Physiology and Chronic Health Evaluation-II; SOFA, Sequential Organ Failure Assessment. ^a^With Mann-Whitney's U-test, between GP patients' group and GN patients' group.

**Figure 2:**
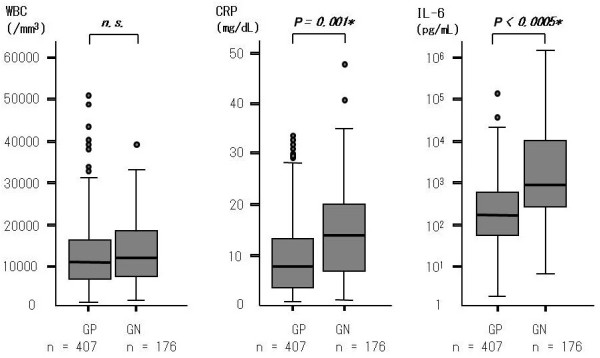
**WBC, CRP and IL-6 levels in GP sample group and GN sample group**. Blood samples used for measurement of laboratory parameters were collected concomitantly with sampling for blood culture. *P value calculated by Student's t-test. CRP, C-reactive protein; GP, gram-positive sample group; GN, gram-negative sample group; IL-6, interleukin-6; WBC, white blood cell count.

The 515 eligible culture-positive blood samples harbored a total of 593 isolates of microorganisms, including 407 isolates of Gram-positive bacteria, 176 isolates of Gram-negative bacteria, and 10 isolates of fungi. Two or more different microbial species were concomitantly detected in 60 blood culture samples. As demonstrated in Figure 2, both CRP and IL-6 blood level were significantly higher in the GN sample group.

## Discussion

We reviewed medical records of septic patients admitted to the ICU and being positive on blood culture during the last eight years for comparison of background characteristics, WBC, CRP, and IL-6 as well as causative microorganisms and clinical outcome. When eligible patients were classified into three groups by severity of sepsis, the prevalence of Gram-negative bacteremia, prevalence of bacteremia caused by both Gram-positive and Gram-negative bacteria, and IL-6 blood level were significantly higher in the septic shock group than in either of the other two groups (Table 1). When episodes of bacteremia caused by Gram-positive and Gram-negative bacteria were compared, CRP and IL-6 blood level were found to be significantly higher in Gram-negative bacteremia (Figure 2). Notably, the sample size in the present study (176 and 407 for episodes of Gram-negative and Gram-positive bacteremia, respectively) is larger than that in any other similar study published to date.

Although differences in the magnitude of insult depending on the type of pathogen, that is, the type of pathogen-associated molecular patterns (PAMPs), have been already recognized [13], few studies have examined this difference quantitatively. While Fisher et al. [14] previously reported that plasma IL-6 levels were significantly higher in patients with Gram-negative bacteremia (n = 17) than in those with Gram-positive bacteremia (n = 12), the present study is, to the best of our knowledge, the first demonstration of such differences in response to bacterial bloodstream infection among different causative bacterial species in a sufficiently large study population. Our finding that CRP and IL-6 blood level were significantly higher in Gram-negative bacteremia than in Gram-positive bacteremia suggests that different types of PAMPs may induce different types and magnitudes of response. Since IL-6 is not only an index of response to invasion but also a typical alarmin [15], IL-6 per se may induce further exacerbation of pathophysiological condition.

The magnitude of biological response to insult has been believed to be determined by the magnitude of insult as well as host predisposition. This concept has been schematized in the recently proposed PIRO model (Predisposition, Insult, Response, and Organ dysfunction) [16]. When the PIRO model is applied to cases of sepsis, the nature of insult can be considered infection, with the site, type, and extent of infection significantly impacting prognosis [16]. Furthermore, it is known that the mechanisms of bacterial virulence vary depending on bacterial species and strain [17]. For example, S. aureus produces protein A, which is a ligand for tumor necrosis factor (TNF) receptor-1 and induces a response identical to that caused by TNF-α stimulation [18]. In addition, some Group B Streptococcus strains produce C5a peptidase to inhibit activation of the complement system [19]. Differences in mechanisms of bacterial virulence result in differences in host response, that is, differences in the extent of activation of various signaling cascades and stimulation/inhibition of host cell apoptosis [17,20], leading to influence prognosis.

Earlier initiation of appropriate antimicrobial therapy is clearly crucial in the treatment of sepsis [21]. On the other hand, though no countermeasures taking differences in the mechanisms of bacterial virulence into account are currently available in clinical practice, antimicrobial therapy beyond conventional antimicrobial medications is urgently needed. Some recent studies suggest future possibilities for such therapies. For example, inhibition of quorum-sensing regulated genes of Pseudomonas aeruginosa by synthetic furanones improved survival in a mouse model of pneumonia [22]. Such virulence-targeting antimicrobial therapies are expected to provide new options for the treatment of sepsis in ICU [23].

PAMPs from Gram-negative and Gram-positive bacteria are known to act as ligands for mutually different pattern recognition receptors including Toll-like receptors [24], and the molecular mechanisms underlying the differential responses to infection with Gram-negative and Gram-positive bacteria have been investigated [25]. However, the effects of differences in the molecular mechanisms of response to invasion of Gram-negative and Gram-positive bacteria on the clinical course and prognosis of sepsis require further clarification. In particular, IL-6 and CRP are known to be relatively non-specific biomarkers, compared with more validated biomarkers for sepsis, such as procalcitonin and triggering receptors expressed on myeloid cell (TREM)-1. The differences of blood levels of such non-specific biomarkers warrant further characterization at the molucular level of the differences in virulence mechanisms between Gram-negative and Gram-positive bacteremia.

The present study has the following limitations. First, it was a retrospective study. Second, it was a single-center study in which it is difficult to rule out the possibility of bias in bacterial species identified and in patients' characteristics. In particular, the high percentage of male patients (69.5%, Table 1) implies such possibility of bias, even though this male-female ratio was consistent with that of all patients admitted to our ICU during eight years (63.3%, n = 4,092, male 2,590, female 1,502). However, the same male predominance in ICU population was also noticed before, even though the reason for this male predominance is unknown [26]. Length of ICU stay also might suggest the existence of bias, since the three groups divided by severity of sepsis did not show differences in length of stay. In addition to that, since patients in the septic shock group and the severe sepsis group were significantly older than the sepsis group patients, those differences might affect the magnitude of inflammatory reactions and outcomes. Regarding the severity of Gram-positive and Gram-negative patient groups, SOFA score did not demonstrate the significant difference between groups, though APACHE II score did (Table 2). This result suggests that the number of patients might not be enough to reach the conclusion. Furthermore, because molecular mechanism of virulence underlying the present findings is not yet clarified, these results cannot be directly translated into practical management. Nevertheless, the present study has the merit of having demonstrated differences in blood cytokine levels in bacteremia caused by different bacterial species in a study population larger than that of any of the other similar studies.

## Conclusions

Patients admitted to the ICU with bacteremia were classified according to severity of sepsis for comparison of pathogenic microorganisms and blood levels of inflammatory biomarkers. The incidence of Gram-negative bacteria and CRP and IL-6 blood level were significantly higher in the septic shock group than in the sepsis and severe sepsis groups. Furthermore, CRP and IL-6 blood level measured concomitantly with sampling for blood culture were significantly higher in Gram-negative bacteremia than in Gram-positive bacteremia. These findings suggest that differences in host responses and virulence mechanisms of different pathogenic microorganisms should be considered in treatment of bacteremic patients, and that new countermeasures beyond conventional antimicrobial medications are urgently needed.

## Key messages

• CRP and IL-6 blood level were significantly higher in Gram-negative bacteremia than in Gram-positive bacteremia.

• The incidence of Gram-negative bacteremia was significantly higher in bacteremic ICU patients with septic shock than in those with sepsis or severe sepsis.

• Characterization at the molecular level of the differences in virulence mechanisms between Gram-negative and Gram-positive bacteria is required.

## Abbreviations

APACHE-II: Acute Physiology and Chronic Health Evaluation-II; CRP: C-reactive protein; IL-6: interleukin-6; PAMPs: pathogen-associated molecular patterns; SOFA: Sequential Organ Failure Assessment; WBC: white blood cell

## Competing interests

The authors declare that they have no competing interests.

## Authors' contributions

RA designed the study and interpreted the results. OS and HH made critical revision of the manuscript for important intellectual content. TS, MN, YH, and YT drafted the manuscript. KS participated in the analysis of data and performed the statistical analysis. All authors read and approved the final manuscript.

## Authors' information

RA is Assistant Professor, Department of Emergency and Critical Care Medicine, Chiba University Hospital and a Board Certified Member of the Japanese Society of Intensive Care Medicine. OS is Professor and Chairman, Department of Emergency and Critical Care Medicine, Chiba University Graduate School of Medicine, and a Board Certified Member of the Japanese Society of Intensive Care Medicine. HH is Professor Emeritus and Former Chairman, Department of Emergency and Critical Care Medicine, Chiba University Graduate School of Medicine, and is Immediate Past President of the Japanese Society of Intensive Care Medicine.

## References

[B1] Martin GS Mannino DM Eaton S Moss M The epidemiology of sepsis in the United States from 1979 through 2000 N Engl J Med 2003 348 1546 1554 10.1056/NEJMoa022139 12700374

[B2] Hotchkiss RS Karl IE The pathophysiology and treatment of sepsis N Engl J Med 2003 348 138 150 10.1056/NEJMra021333 12519925

[B3] Bearman GM Wenzel RP Bacteremias: a leading cause of death Arch Med Res 2005 36 646 659 10.1016/j.arcmed.2005.02.005 16216646

[B4] Renaud B Brun-Buisson C Outcomes of primary and catheter-related bacteremia. A cohort and case-control study in critically ill patients Am J Respir Crit Care Med 2001 163 1584 1590 11401878 10.1164/ajrccm.163.7.9912080

[B5] Digiovine B Chenoweth C Watts C Higgins M The attributable mortality and costs of primary nosocomial bloodstream infections in the intensive care unit Am J Respir Crit Care Med 1999 160 976 981 10471627 10.1164/ajrccm.160.3.9808145

[B6] Annane D Aegerter P Jars-Guincestre MC Guidet B Current epidemiology of septic shock: the CUB-Rea Network Am J Respir Crit Care Med 2003 168 165 172 10.1164/rccm.2201087 12851245

[B7] Garrouste-Orgeas M Timsit JF Tafflet M Misset B Zahar JR Soufir L Lazard T Jamali S Mourvillier B Cohen Y De Lassence A Azoulay E Cheval C Descorps-Declere A Adrie C Costa de Beauregard MA Carlet J OUTCOMEREA Study Group Excess risk of death from intensive care unit-acquired nosocomial bloodstream infections: a reappraisal Clin Infect Dis 2006 42 1118 1126 10.1086/500318 16575729

[B8] Wisplinghoff H Bischoff T Tallent SM Seifert H Wenzel RP Edmond MB Nosocomial bloodstream infections in US hospitals: analysis of 24,179 cases from a prospective nationwide surveillance study Clin Infect Dis 2004 39 309 317 10.1086/421946 15306996

[B9] Oda S Hirasawa H Shiga H Nakanishi K Matsuda K Nakamua M Sequential measurement of IL-6 blood levels in patients with systemic inflammatory response syndrome (SIRS)/sepsis Cytokine 2005 29 169 175 10.1016/j.cyto.2004.10.010 15652449

[B10] American College of Chest Physicians/Society of Critical Care Medicine Consensus Conference: definitions for sepsis and organ failure and guidelines for the use of innovative therapies in sepsis Crit Care Med 1992 20 864 874 1597042

[B11] Knaus WA Draper EA Wagner DP Zimmerman JE APACHE II: a severity of disease classification system Crit Care Med 1985 13 818 829 10.1097/00003246-198510000-00009 3928249

[B12] Vincent JL de Mendonca A Cantraine F Moreno R Takala J Suter PM Sprung CL Colardyn F Blecher S Use of the SOFA score to assess the incidence of organ dysfunction/failure in intensive care units: results of a multicenter, prospective study. Working group on "sepsis-related problems" of the European Society of Intensive Care Medicine Crit Care Med 1998 26 1793 1800 9824069 10.1097/00003246-199811000-00016

[B13] Lotze MT Zeh HJ Rubartelli A Sparvero LJ Amoscato AA Washburn NR Devera ME Liang X Tor M Billiar T The grateful dead: damage-associated molecular pattern molecules and reduction/oxidation regulate immunity Immunol Rev 2007 220 60 81 10.1111/j.1600-065X.2007.00579.x 17979840

[B14] Fisher CJ Jr Opal SM Dhainaut JF Stephens S Zimmerman JL Nightingale P Harris SJ Schein RM Panacek EA Vincent JL Foulke GE Warren EL Garrard C Park G Bodmer MW Cohen J der Linden CV Cross AS Sadoff JC The CB0006 Sepsis Syndrome Study Group Influence of an anti-tumor necrosis factor monoclonal antibody on cytokine levels in patients with sepsis. The CB0006 Sepsis Syndrome Study Group Crit Care Med 1993 21 318 327 8440099 10.1097/00003246-199303000-00006

[B15] Oppenheim JJ Yang D Alarmins: chemotactic activators of immune responses Curr Opin Immunol 2005 17 359 365 10.1016/j.coi.2005.06.002 15955682

[B16] Rubulotta F Marshall JM Ramsay G Nelson D Levy M Williams M Predisposition, insult/infection, response, and organ dysfunction: A new model for staging severe sepsis Crit Care Med 2009 37 1329 1335 10.1097/CCM.0b013e31819d5db1 19242329

[B17] Webb SA Kahler CM Bench-to-bedside review: Bacterial virulence and subversion of host defences Crit Care 2008 12 234 10.1186/cc7091 19014410 PMC2646333

[B18] Gomez MI Lee A Reddy B Muir A Soong G Pitt A Cheung A Prince A Staphylococcus aureus protein A induces airway epithelial inflammatory responses by activating TNFR1 Nat Med 2004 10 842 848 10.1038/nm1079 15247912

[B19] Jarva H Jokiranta TS Wurzner R Meri S Complement resistance mechanisms of streptococci Mol Immunol 2003 40 95 107 10.1016/S0161-5890(03)00108-1 12914816

[B20] Finlay BB McFadden G Anti-immunology: evasion of the host immune system by bacterial and viral pathogens Cell 2006 124 767 782 10.1016/j.cell.2006.01.034 16497587

[B21] Ibrahim EH Sherman G Ward S Fraser VJ Kollef MH The influence of inadequate antimicrobial treatment of bloodstream infections on patient outcomes in the ICU setting Chest 2000 118 146 155 10.1378/chest.118.1.146 10893372

[B22] Wu H Song Z Hentzer M Andersen JB Molin S Givskov M Hoiby N Synthetic furanones inhibit quorum-sensing and enhance bacterial clearance in Pseudomonas aeruginosa lung infection in mice J Antimicrob Chemother 2004 53 1054 1061 10.1093/jac/dkh223 15117922

[B23] Clatworthy AE Pierson E Hung DT Targeting virulence: a new paradigm for antimicrobial therapy Nat Chem Biol 2007 3 541 548 10.1038/nchembio.2007.24 17710100

[B24] Akira S Uematsu S Takeuchi O Pathogen recognition and innate immunity Cell 2006 124 783 801 10.1016/j.cell.2006.02.015 16497588

[B25] Elson G Dunn-Siegrist I Daubeuf B Pugin J Contribution of Toll-like receptors to the innate immune response to Gram-negative and Gram-positive bacteria Blood 2007 109 1574 1583 10.1182/blood-2006-06-032961 17038528

[B26] Sirio CA Tajimi K Tase C Knaus WA Wagner DP Hirasawa H Sakanishi N Katsuya H Taenaka N An initial comparison of intensive care in Japan and the United States Crit Care Med 1992 20 1207 1215 10.1097/00003246-199209000-00006 1521435

